# Research on the optimization of financing scheme of long-term care insurance in China based on system dynamics simulation

**DOI:** 10.3389/fpubh.2022.908864

**Published:** 2022-09-23

**Authors:** Liangwen Zhang, Sijia Fu, Yifan Wang, Ya Fang

**Affiliations:** ^1^State Key Laboratory of Molecular Vaccinology and Molecular Diagnostics, School of Public Health, Xiamen University, Xiamen, China; ^2^Key Laboratory of Health Technology Assessment of Fujian Province University, School of Public Health, Xiamen University, Xiamen, China; ^3^School of Public Health, Li Ka Shing Faculty of Medicine, The University of Hong Kong, Pok Fu Lam, Hong Kong SAR, China

**Keywords:** long-term care insurance, financing mechanism, system dynamic model, simulation and optimization, sustainability

## Abstract

**Objective:**

The aging population in China highlights the significance of long-term care insurance (LTCI). This study provides policy suggestions for China to establish a sustainable LTCI financing mechanism by predicting the trend of funds balance and screening the appropriate financing scheme.

**Method:**

A system dynamics model (SDM) of LTCI funds was constructed by clarifying the current situation and its main influencing factors of revenue and expenditure of LTCI funds in China. Also, through literature research and expert consultation, we found the intervention goals and predicted the changing trend of LTCI fund balance from 2020 to 2050 under different intervention schemes.

**Results:**

The SDM of LTCI financing passes the dimensional consistency test, structural test, and historical test. Therefore, it can objectively reflect the structure and behavior of the financing system. It is found that the factors affecting the revenue and expenditure system of LTCI funds mainly include economic factors, demographic factors, and other factors. By adjusting three intervention indicators, namely, individual payment rate, reimbursement proportion, and severe disability rate, this study produces 45 financing combination schemes. By comparing the changing trend of LTCI fund balance under different intervention schemes, according to the screening principle, five better financing schemes are finally selected. These five financing schemes have no deficit and excessive balance in the forecast period, which is in line with the principle of sustainability. It can provide a reference for the selection of financing schemes in pilot areas.

**Discussion:**

This study has optimized the policy of the LTCI financing mechanism, determined the suitable LTCI participants, financing channels and levels, and screened out the suitable LTCI financing policy optimization scheme for China. By appropriately increasing the individual payment rate, strengthening the disability intervention of the elderly, formulating scientific and objective disability evaluation standards, and finally establishing a dynamic financing adjustment mechanism of LTCI. This study can provide a basis for the scientific formulation of the LTCI financing mechanism in China and provide a reference for developing countries to establish a sustainable LTCI.

## Introduction

Population aging has become a serious social problem faced by many countries (1). As a developing country with the largest elderly population in the world, China faces the enormous pressure of an aging population (2). Especially in the background of novel coronavirus pneumonia, policymakers realized that the trend of population aging has brought a great impact on the current medical and health system in China. Therefore, how to further improve the development of the social security system is particularly important (3). The results of the seventh national census show that there are 260 million people aged 60 and over in China, accounting for 18.7% of the total population (4). It is predicted that by 2050, the elderly population over 60 will be nearly 500 million, accounting for one-third of the total population. The cost of LTC has brought economic and nursing burdens to the disabled elderly and their families, which means that more public expenditure is needed (5).

The disease spectrum in China is accelerating from infectious diseases to noncommunicable diseases. Chronic noncommunicable diseases are the main cause of death and disability in the elderly. It is predicted that by 2050, the total number of disabled elderly in China will rapidly increase from 43.75 million in 2020 to 91.4 million in 2050 (6). The one-child policy implemented for a long time in China has led to a small family structure and a large proportion of empty nesters, which has exacerbated the care pressure on the disabled elderly. Most of the elderly have a low income, which is difficult to support the long-term high nursing expenses, which brings a heavy economic burden to the family and is not conducive to the harmonious and stable development of society (7). Therefore, China urgently needs to introduce unified long-term care insurance (LTCI) system to reduce the huge care and economic burden faced by disabled families.

In 2016, the Ministry of Human Resources and Social Security of the People's Republic of China issued the “Guideline on Carrying out an LTCI System Pilot” and began to explore the LTCI system in 15 cities in China (8). In May 2020, the National Healthcare Security Administration issued guidance on expanding the pilot of the LTCI system, adding 14 pilot cities to further promote the exploration and development of LTCI (7). However, due to the different levels of social and economic development in various regions, the degree of aging and the supply level of elderly care services are quite different, and China has not yet formed a unified LTCI system. LTCI participation is related to an individual's Social Medical Insurance (SMI) status (3). China currently has two SMI schemes: the urban employee SMI, covering urban residents with formal employment, and the urban and rural resident's SMI covering rural and urban residents without formal employment. The insured of LTCI must be the insured of the urban employee insurance or the urban and rural residents' medical insurance (5). Among them, financing is not only the core of establishing LTCI but also the basis for the formation of insurance funds. It mainly includes the insured object, financing source, financing channel, and financing level (7). At present, LTCI in most regions mainly relies on the fund's balance of SMI, so it lacks independence (3). Depending on SMI, it will be difficult to define the responsibility subjects of the two social insurance schemes. In the long run, it will lead to a surge of operation and management pressure on the two social insurance schemes and even confusion of fund accounts. LTCI will be difficult to implement and will have a certain negative impact on the operation of SMI (5).

At present, foreign scholars' research on the financing of LTCI is more comprehensive. Among them, some studies compare the formulation of LTCI financing mechanism policies in different developed countries (9–12) and provide suggestions for the formulation of LTCI financing mechanisms in developing China (13). Others discussed long-term care (LTC) cost calculation (14), cost control (5), and optimization of the financing model (15–17). At the same time, foreign scholars introduced the system dynamics model (SDM) to study the social security system (16). For example, Nobuo Nishi explored the relationship between LTC needs and medical costs by establishing SDM (18). Few have studied the financing mechanism of LTCI from a systematic perspective. SDM is a simulation modeling method used to represent the structure of complex systems (19, 20). It provides a unique quantitative model for policy analysis (21). It first puts forward the causal hypothesis, generates the causal relationship diagram, and then transforms the qualitative hypothesis into a quantitative simulation to test the policy effect. Policymakers can analyze the experimental mode and trend through the simulation results, which provides a basis for decision-making (22, 23).

Long-term care insurance in China started late and has not yet introduced a unified national LTCI system. Due to the lack of data and the primary stage of the development of LTCI, most studies refer to the LTCI policies of developed countries to put forward policy suggestions on the Chinese LTCI system (24–26). Although the experience and insights of these developed countries are valuable, China needs to develop its own LTCI financing mechanism according to its own cultural and economic development levels. At the same time, some studies have assessed the sustainability of LTCI financing (27, 28) but have not combed and evaluated the LTCI system as a whole, and it is of great significance to explore the impact of different strategic scenarios on the LTCI insurance financing system.

Therefore, this study takes the LTCI financing mechanism as the research object and constructs the SDM of LTCI financing in China. First, the SDM of the LTCI financing system is established by clarifying the revenue and expenditure of LTCI funds and its main influencing factors. Second, the changing trend of LTCI funds is simulated within the specified period (2020–2050). Finally, based on the perspective of fairness, efficiency, and sustainability, this study systematically evaluates the revenue, expenditure, and balance of the LTCI funds under different financing schemes and further selects the optimization strategy of LTCI financing.

This study not only provides optimization scheme suggestions for the financing of Chinese LTCI funds but also provides a reference for developing countries to establish a sustainable LTCI financing mechanism.

## Materials and methods

### Data sources

Data for this study are taken from field research, expert consultation, China Statistical Yearbook, and published research literature. Based on the principle of typical sampling, four representative LTCI pilot cities (Jiaxing, Shanghai, Jingmen, and Chengdu) were selected from the east, middle, and west of China to conduct the field research in August 2020. Due to the differences in economic development and population structure, the four pilot cities are actively exploring LTCI financing mechanisms that meet their own actual conditions and have formed a relatively complete policy system, such as the similarities and differences of the insured population, the protection objects, the types of care, etc., which provides a reference for the further development of LTCI in China. Through the questionnaire survey and interviews with government officials, heads of commercial insurance, and nursing service institutions, information on the implementation of LTCI in the pilot cities was collected through the questionnaire. A total of 11 questionnaires were distributed, and all were effectively recovered. On the other hand, we interviewed 30 relevant personnel, focusing on the insured population, financing methods and levels, disability rate, etc., to reflect the overall construction of the LTCI financing mechanism. At the same time, this study mainly focuses on the analysis of the LTCI policy. Some sensitive information, such as name, age, and ID number, has not been collected. The main data sources are listed in Supplementary Table 1.

### Key assumptions

At present, China has not implemented a unified LTCI system, and each pilot city has carried out the corresponding exploration according to the local actual situation. Therefore, according to the published research literature and field research, the main assumptions of this study are as follows: (a) it is assumed that 2019 is the base period and the forecast period is 2020–2050; (b) at present, the enrollment in LTCI is linked to the SMI status of individuals in pilot cities. To ensure the fairness of the implementation of the system, it is assumed that those who participate in LTCI are the insured of all SMI, and those who enjoy the benefits of LTCI are the severely disabled people in the insured population; (c) LTCI funds mainly come from premium payments, without taking into account the investment income; and (d) the economic development is relatively stable, and the per capita GDP and wage levels maintain a certain growth rate without major fluctuations.

### Model construction

In this paper, through field research, expert consultation, and consulting the relevant literature on LTCI, the LTCI financing system is defined as two subsystems, namely, the LTCI fund revenue subsystem and the expenditure subsystem. Among them, the revenue subsystem of the LTCI funds includes the revenue subsystem of urban employees, the revenue subsystem of retired employees, and the revenue subsystem of urban and rural residents. Based on the purpose of the study and the actual operation of LTCI financing, this study makes a loop analysis of the revenue subsystem and expenditure subsystem of the LTCI funds. The variables of the two subsystems and each subsystem are interrelated and restricted and ultimately affect the balance of the LTCI funds (Supplementary Figure 1). In this figure, the arrow represents the relationship among variables, and the direction of each line shows the direction of the effect. The sign “+” dictates that the variables change in the same direction, while the sign “-” is the opposite (18).

**Figure 1:**
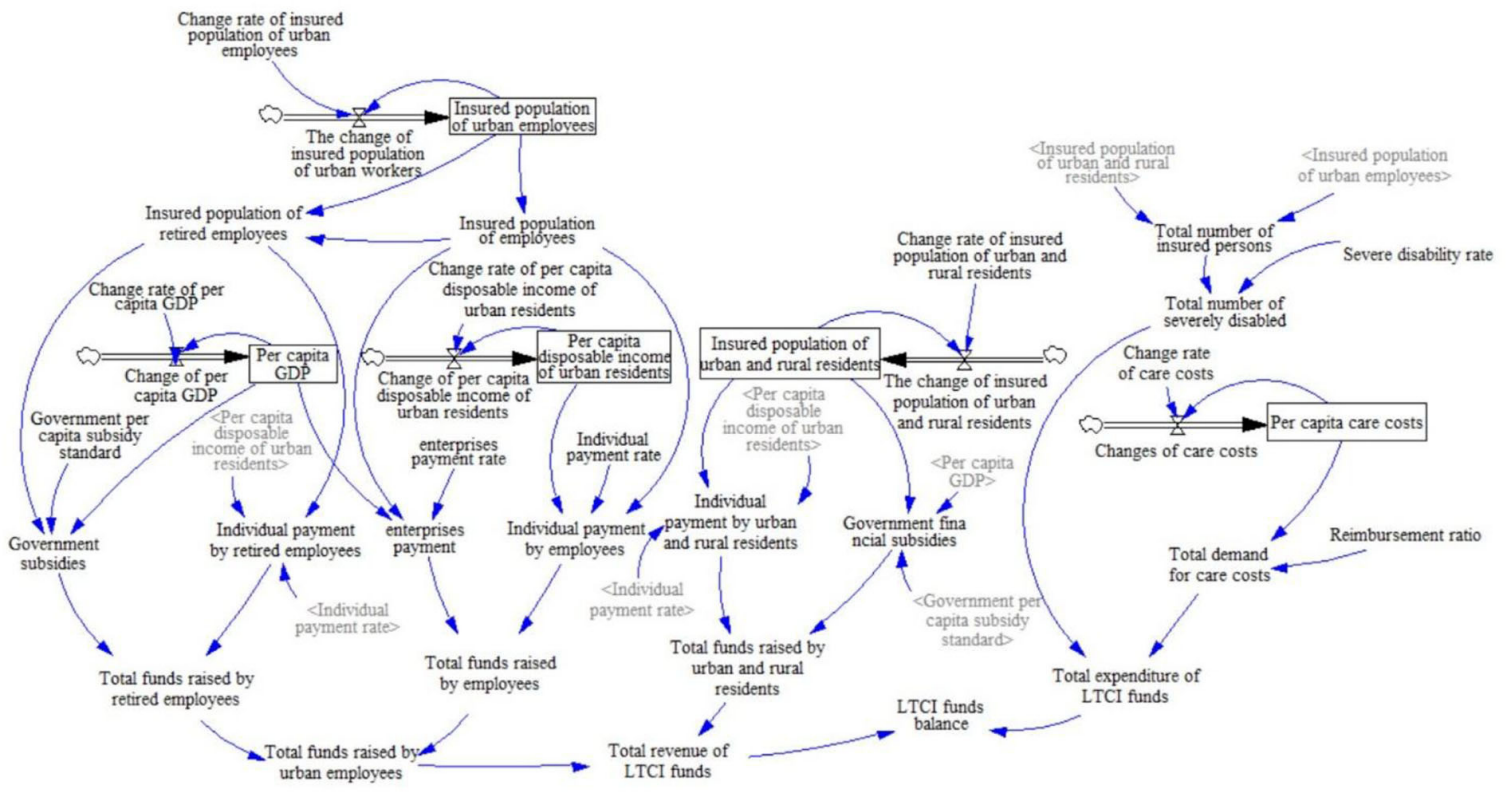
Flow diagram of long-term care insurance (LTCI) financing system.

According to the causality diagram, combined with literature analysis, regression analysis, and actuarial methods, the system state variables, rate variables, auxiliary variables, and constants are quantitatively analyzed, parameters are estimated (Supplementary Table 2), and the LTCI financing SDM is constructed (Figure 1). Among them, the regression analysis is used to determine whether there is a quantitative relationship between the insured population of employees and the insured population of urban employees, and the corresponding relationship equation is constructed. The state variable represents the cumulative effect of the system, which means that the current cumulative value is equal to the original value plus the change amount. The rate variable represents the amount by which the accumulation effect changes quickly or slowly. In addition, the auxiliary variable is the amount of information in the system, which refers to the intermediate variable from the state variable to the rate variable. Among them, the construction of causality diagrams and models is established by using the Vensim DSS software.

### Validation of the model

#### Sensitivity analysis

Based on the LTCI operation situation, combined with field investigations, we selected three key variables that affect the revenue and expenditure of the LTCI funds for sensitivity analysis, including individual payment rate, reimbursement ratio of nursing service, and severe disability rate, to verify the influence of parameters on the balance of LTCI funds to achieve sensitivity analysis. We set the number of verifications to 200 and use a random uniform distribution for verification. The results show that by adjusting the parameter value range, the LTCI fund balance has changed significantly (Supplementary Figure 2), which provides a reference for policy intervention.

Therefore, through the summary and analysis of literature and survey data, this study divides the individual payment rate into three situations: 0.08, 0.09, and 0.1% (approximately Chinese ¥34–43 per person per year). When the individual payment rate is fixed, the severe disability rate was divided into three cases of 0.25, 0.3, and 0.35%, and the reimbursement rates of 0.7, 0.75, 0.8, 0.85, and 0.9 were classified and discussed (22).

#### Historical test

The study constructed the SDM of LTCI financing through the dimensional consistency test, the structure test, and the historical test. According to the availability of data, the historical test simulation data select the number of urban employees insured by the SMI in demographic factors and the per capita disposable income of urban residents in economic factors for comparative analysis with the data of urban employees insured by the SMI and per capita disposable income of urban residents in China Statistical Yearbook from 2011 to 2019. The test results are shown in Supplementary Table 3. The average errors between the actual data and the simulated data are 0.78 and −3.41% (both <10%), respectively. Within a reasonable range, it shows that the model has a high fitting degree, is effective, and is reasonable (29).

### Criteria for evaluating financing scheme

The simulated fund balance of an appropriate financing plan should be neither deficit nor excessive. A deficit would mean that the fund would not be able to meet social needs in the future under this financing scheme. The excessive balance indicates that the contribution burden is too heavy, and the fund use efficiency is low (30), which is not a good financing scheme. The change in fund balance is closely related to the national economy. If the fluctuation is too obvious, it will cause a great burden and unknown risk to the social economy. Therefore, the better financing scheme is that the fund balance simulated by the model presents a relatively flat curve no lower than the horizontal axis, and the highest value (inflection point) should appear in the later period of the forecast year.

According to the literature and information on government websites, combined with the pension and SMI fund balance statistics in recent years, this study considers the fund balance of more than Chinese ¥3 trillion to be excessive. Therefore, all financing schemes with deficits or excessive balances during the forecast period are considered unreasonable financing schemes. The simulation results of all schemes are as follows.

## Results

### Change trend of fund balance

#### Simulation results and analysis of each combination scheme when the individual payment rate is 0.08%

When the individual payment rate is 0.08%, it is combined with the severe disability rate and the reimbursement proportion of LTCI according to the set value. The cumulative balance of LTCI funds from 2020 to 2050 is shown in Supplementary Tables 4.1–4.3. When the individual payment rate is 0.08% and when the severe disability rate is 0.25%, the two schemes with reimbursement ratios of 0.7 and 0.75 predict that the fund balance is too much, more than Chinese ¥300 billion, so it is not advisable. When the severe disability rate is 0.3%, the two schemes with reimbursement rates of 0.85 and 0.9 have deficits in the fund balance at the later stage of the forecast, which is also undesirable. When the severe disability rate is 0.35% and the reimbursement rate is 0.75, 0.8, 0.85, and 0.9, the four schemes show a fund balance deficit in the later prediction period, which is not desirable.

**Figure 2:**
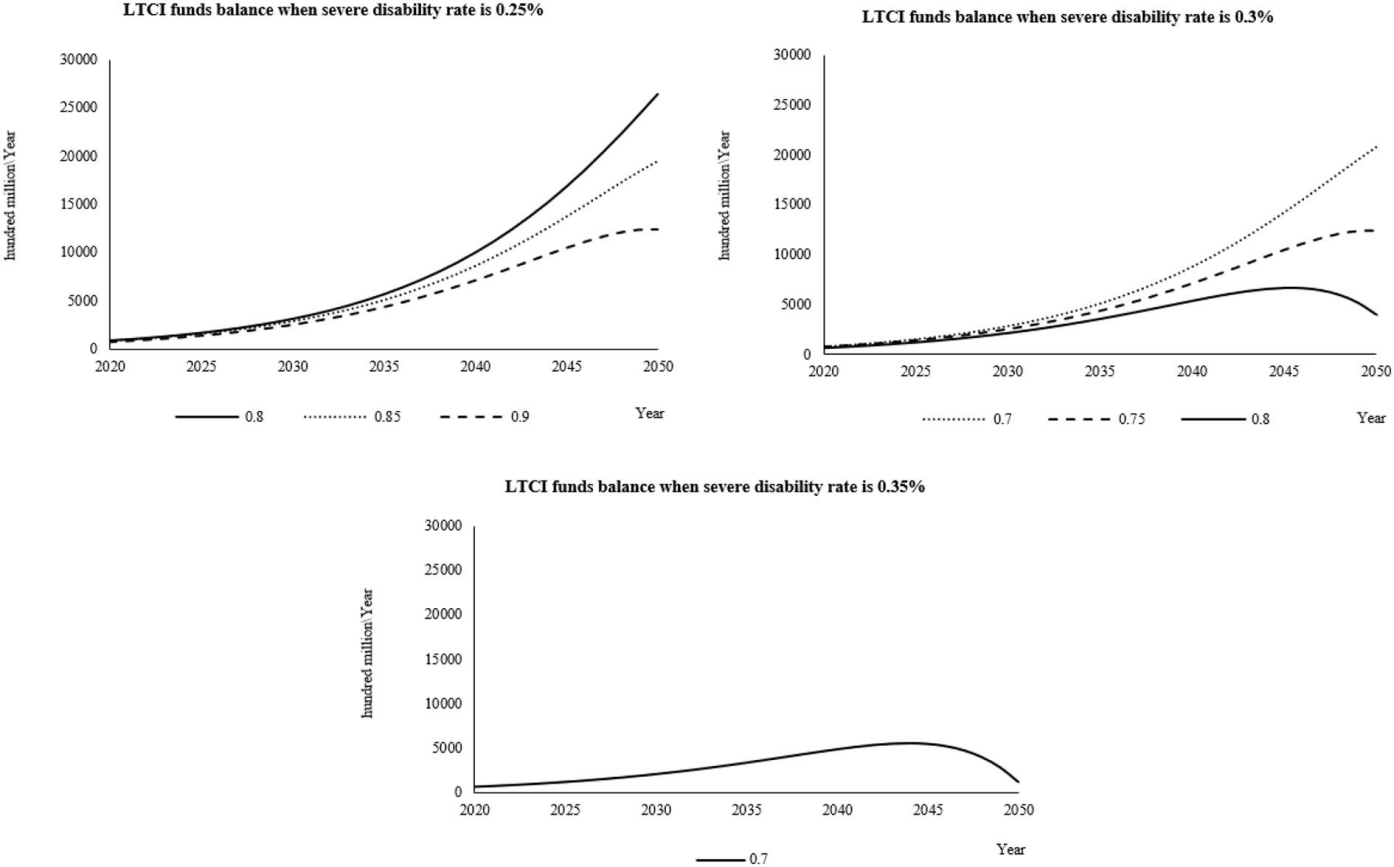
When the individual payment rate is 0.08%, the fund balance changes in the reasonable financing scheme of LTCI under different severe disability rates.

Combined with the curve changes (Figure 2), when the severe disability rate is 0.25%, the reimbursement ratio is 0.9, and when the severe disability rate is 0.3%, the reimbursement ratio is 0.75 and 0.8, respectively. These three schemes have the highest values in the later stage of the forecast, so it can better explain that the fund balance is at a more reasonable value in the forecast period. When the severe disability rate is 0.25% and the reimbursement ratio is 0.7, it can be seen from the change chart that there will be a balance deficit, which is more unreasonable than other schemes.

#### Simulation results and analysis of each combination scheme when the individual payment rate is 0.09%

When the individual payment rate is 0.09%, it is combined with the severe disability rate and the reimbursement proportion of LTCI according to the set value. The cumulative balance of LTCI funds from 2020 to 2050 is shown in Supplementary Tables 5.1–5.3. When the individual payment rate is 0.09% and when the severe disability rate is 0.25%, the reimbursement ratio is 0.7, 0.75, and 0.8. These three schemes have too much fund balance in the later prediction period, so they are not desirable. When the severe disability rate is 0.3%, the reimbursement ratio is 0.85 and 0.9, the severe disability rate is 0.35%, and the reimbursement ratio is 0.75, 0.8, 0.85, and 0.9. These six schemes all have balance deficits at the end of the forecast period, which is unsustainable, so it is not advisable.

**Figure 3:**
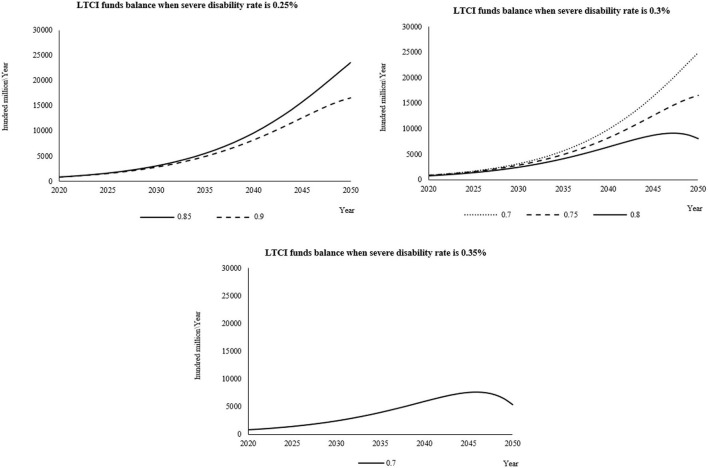
When the individual payment rate is 0.09%, the fund balance changes in the reasonable financing scheme of LTCI under different severe disability rates.

From the curve change chart (Figure 3), when the individual payment rate is 0.09%, the severe disability rate is 0.3%, the reimbursement rate is 0.8, the severe disability rate is 0.35%, and the reimbursement rate is 0.7. The balance under the two schemes has the highest value in the predicted life, which is in line with the better curve change trend set in the study.

#### Simulation results and analysis of each combination scheme when the individual payment rate is 0.1%

When the individual payment rate is 0.1%, it is combined with the severe disability rate and the reimbursement proportion of LTCI according to the set value. The cumulative balance of LTCI funds from 2020 to 2050 is shown in Supplementary Tables 6.1–6.3. When the individual payment rate is 0.1% and when the severe disability rate is 0.25%, the reimbursement ratio is 0.7, 0.75, and 0.8. The three schemes have too much fund balance in the later prediction period, which is not desirable. When the severe disability rate is 0.3% and the reimbursement ratio is 0.9, the fund balance presents a deficit at the end of the forecast period, which is not desirable. Similarly, when the severe disability rate is 0.35%, the reimbursement ratio of 0.75, 0.8, 0.85, and 0.9 are unsustainable financing schemes, so they are also not desirable.

**Figure 4:**
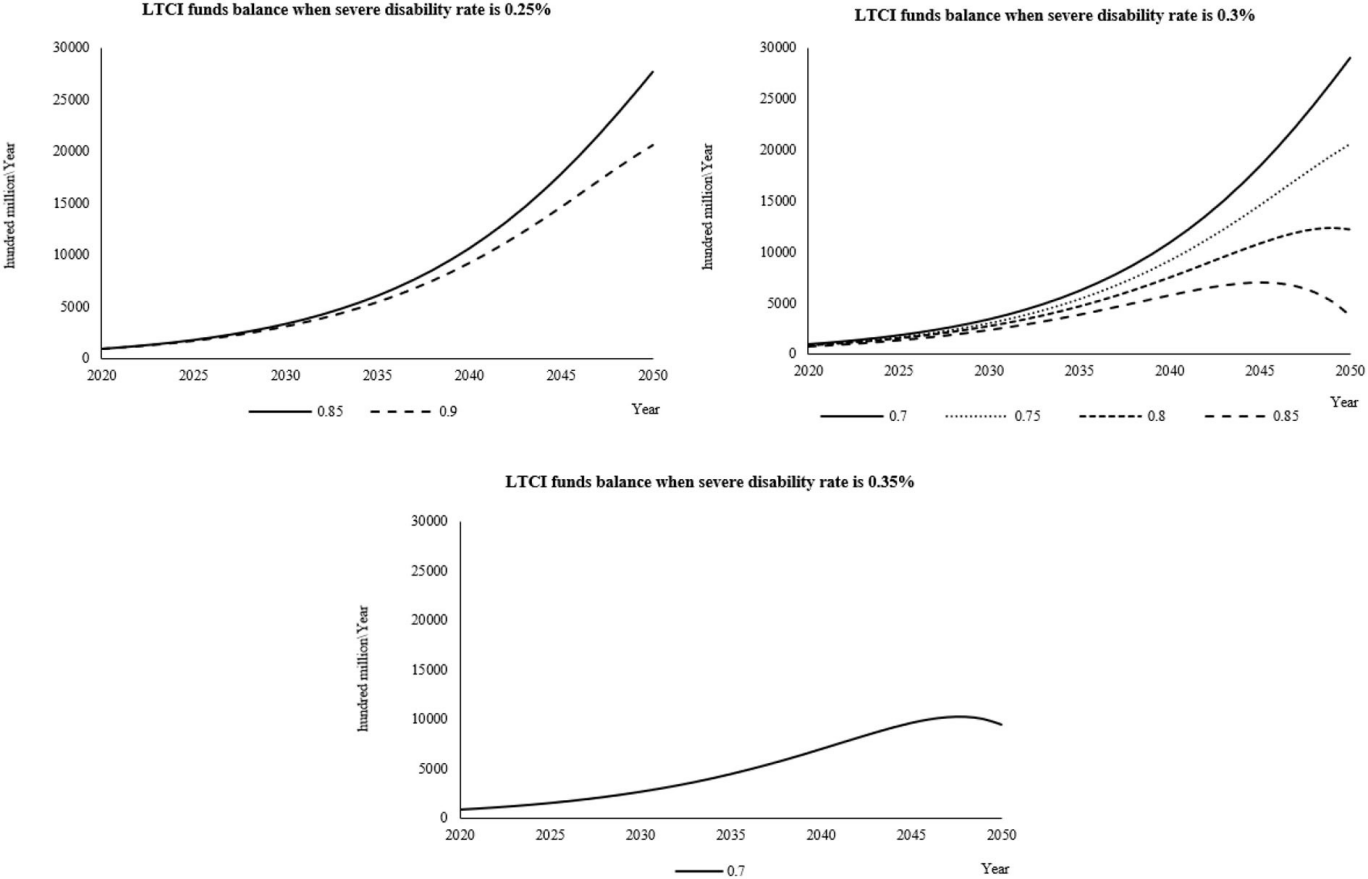
When the individual payment rate is 0.2%, the fund balance changes in the reasonable financing scheme of LTCI under different severe disability rates.

From the change of prediction curve (Figure 4), when the severe disability rate is 0.25%, the reimbursement ratio is 0.85 and 0.9; when the severe disability rate is 0.3%, the reimbursement ratio is 0.7 and 0.75, and the fund balance changes under the four schemes only show an increasing trend in the forecast period. According to the setting of this study, these four schemes are not good financing schemes. On the contrary, when the severe disability rate is 0.3%, the reimbursement ratio is 0.8 and 0.85, the severe disability rate is 0.35%, the reimbursement ratio is 0.7, and there is an inflection point in the change of fund balance under the three financing schemes, which is more reasonable and a good financing scheme.

### Assumption of the appropriate scheme

According to the policy suggestions given in the blue book of healthcare in 2020 (31), it is suggested that the reimbursement level of LTCI is 80–90%. Based on the reasonable scheme selected in combination with the results, the following five financing schemes for LTCI are screened again and considered to be better schemes (Table 1). The curve of the simulated fund balance under these five financing schemes is flat, and there is no deficit in the forecast period and no excessive balance, which conforms to the sustainable principle. It can provide a reference for the selection of financing schemes in pilot areas.

**Table 1:** Assumption of the appropriate scheme.

	**Individual payment rate**	**Severe disability rate**	**Reimbursement ratio**	**Maximum/year**	**Funds balance in 2050**
	**(%)**	**(%)**	**(%)**	**(Billion yuan)**	**(Billion yuan)**
1	0.08	0.25	90	12,440/2,050	12,440
2	0.08	0.3	80	6,693/2,046	4,020
3	0.09	0.3	80	9,122/2,048	8,119
4	0.1	0.3	80	12,360/2,049	12,210
5	0.1	0.3	85	6,957/2,046	3,796

## Discussion

The financing system of LTCI involves complex multidimensional variables such as social demography, health management, and economics. This study analyzes the main influencing factors of the LTCI financing mechanism, constructs the SDM of LTCI funds financing in China, and tests the effectiveness of the model. Furthermore, this study selects the intervention experimental group, uses the control variable method, controls the key variables in turn, and compares the changing trend of LTCI funds balance from 2020 to 2050 under different intervention schemes. This study fully considers the interaction between various factors and subsystems in the system and can truly reflect the operation of fund revenue and expenditure. The research results have reference value.

It is found that the factors affecting the revenue and expenditure system of LTCI funds mainly include economic factors, demographic factors, and other factors, including the per capita disposable income of urban residents, the number of urban employees participating in medical insurance, nursing willingness, etc. The subjects involved include the government, enterprises, insured persons, nursing institutions, etc.

First, when the individual payment rate and severe disability rate are fixed, the accumulated balance of LTCI funds will gradually decrease with the increase of reimbursement proportion, and there is a negative correlation between them. The increase in reimbursement ratio led to the increase of LTCI fund expenditure and accelerated the deficit of fund balance. The “Guideline on Carrying out an LTCI System Pilot”(8) pointed out that differentiated treatment guarantee policies should be formulated according to the level of care and the mode of service provision, and the fund payment level should be controlled at approximately 70% for the LTC expenses that meet the regulations. Therefore, to maintain the sustainability of the scale of the funds, the initially set payment standard for nursing services should not be too high and should be reasonably determined according to the principle of “determining expenditure by income, balancing revenue and expenditure, and a slight balance”.

In addition, when the severe disability rate and reimbursement ratio are fixed, with the increase of individual payment rate, the LTCI fund balance also gradually increases, showing a positive correlation. Therefore, it is an important measure to establish an LTCI financing mechanism borne by individuals, enterprises, and the government. The financing mechanism of LTCI needs to clarify the financing responsibilities of individuals, enterprises, and government financial subsidies. The financing of LTCI can refer to SMI. The financing sources of the insured participating in the SMI for urban employees are composed of individual payment and enterprise payment. The government can give corresponding financial subsidies to the financing of LTCI for urban and rural SMI-insured people based on individual payments (32). When formulating the financing policy of LTCI, policymakers should fully study and consider social factors such as population, economy, and culture and comprehensively consider various indicators in the revenue and expenditure of LTCI, such as LTC expenses and individual and enterprise payment capacity, to ensure that LTCI is a social policy of practical sustainable development. According to the payment level of the existing 15 pilot cities of LTCI, the individual payment amount is in the range of Chinese ¥10–90/year (22). Policymakers to raise a certain level of personal payment within a reasonable range, on the one hand, will help the sustainable operation of LTCI, and on the other hand, it can also reduce the moral hazard in social insurance to a certain extent and help the social masses to improve the prevention awareness of disability (33, 34).

Finally, when the reimbursement ratio and individual payment rate are fixed, the cumulative balance of LTCI funds will gradually decrease with the increase in the severe disability rate, and there is a negative correlation between them. At present, the domestic assessment tools for disability levels are mainly based on the Barthel index, which measures basic daily living ability. The scale is a one-dimensional scale, which mainly takes the basic physical function as the main evaluation index, which may cause deviation between the actual and the consideration results (24). For example, the disability evaluation level of the evaluation object due to paying too much attention to its own behavioral function is more serious than the actual situation, which may lead to a high rate of severe disability (35). Therefore, how to scientifically evaluate the disability level is particularly important. Combined with the current practice in the pilot areas, it is suggested to consider the corresponding disability assessment information management system developing when building the disability assessment tool, avoid subjective and human factors in the assessment, and rely on local medical institutions at all levels to establish the assessment expert database to ensure the fairness and fairness of the assessment. In addition, from the perspective of prevention, we should strengthen disability intervention for the elderly, reduce the rate of severe disability, and more effectively alleviate the pressure on social security (36).

At present, the high demand for LTC services and projects poses a severe challenge in China. LTCI is a social insurance system that provides basic care security for the disabled elderly. It is urgent to establish a unified independent LTCI system in China. In this study, the SDM is applied to optimize the financing mechanism of LTCI. The control variable method is used to control the key variables. The changing trend of LTCI fund balance under different intervention schemes is compared, and the optimization scheme of the LTCI financing mechanism is developed and improved. Some data come from field research in Jiaxing, Shanghai, Chengdu, and Jingmen, which increase the reliability of the model and enrich the quantitative research on the financing optimization of LTCI. However, there are still some limitations to this study. First, this study is based on the national data for simulation analysis and does not analyze the situation according to the economic and cultural differences in different regions, such as urban and rural areas, but the conclusion of this study can be used as the research path for formulating LTCI policies in various regions in the future. In addition, although this study establishes the SDM of the LTCI financing system based on practice, there are still many practical factors that have not been considered and intervened, such as enterprise payment rate and government financial subsidies. Therefore, the development of LTCI in China needs further research and explores a more diversified dynamic adjustment mechanism.

## Conclusion

To sum up, the SDM of the LTCI financing system constructed in this study can objectively reflect the structure and behavior of the LTCI financing system. The results show that by improving the individual payment rate, reasonably determining the reimbursement proportion, and screening the appropriate financing scheme. This study not only provides optimization scheme suggestions for the financing of Chinese LTCI funds but also provides a reference for developing countries to establish a sustainable LTCI financing mechanism. Therefore, in the future, we can timely increase the government's financial subsidies for LTCI for urban and rural residents by establishing a financing mechanism for individuals, enterprises, and the government, thus improving the payment capacity of funds. At the same time, we should build a safeguard mechanism focusing on prevention to ensure the sustainability of the operation of the system.

## Data availability statement

The original contributions presented in the study are included in the article/Supplementary material, further inquiries can be directed to the corresponding author.

## Author contributions

LZ, SF, YW, and YF worked together. Specifically, LZ and SF contributed to the study conception, design, and drafted the manuscript. SF and YF participated in the statistical analysis and drafted the manuscript. YF supervised and revised the manuscript. All authors read and approved the final manuscript.

## Funding

This study was supported by the National Natural Science Foundation of China (Grant No. 81973144), Youth Project of High-end Science and Technology Innovation Think Tank in 2021 (Grant No. 2021ZZZLFZB1207146), and General Project of Xiamen Social Science Research Project in 2022 (Grant No. [2022]C1701). The funders who supported this study had no role in study design, data collection, and analysis, decision to publish, or preparation of the manuscript.

## Conflict of interest

The authors declare that the research was conducted in the absence of any commercial or financial relationships that could be construed as a potential conflict of interest.

## Publisher's note

All claims expressed in this article are solely those of the authors and do not necessarily represent those of their affiliated organizations, or those of the publisher, the editors and the reviewers. Any product that may be evaluated in this article, or claim that may be made by its manufacturer, is not guaranteed or endorsed by the publisher.

## Abbreviations

LTCI: long-term care insurance
SDM: system dynamics model
SMI: Social Medical Insurance
LTC: long-term care.
