# Supplementary material for: Research on the optimization of financing scheme of long-term care insurance in China based on system dynamics simulation
Source: Front Public Health. 2022 Sep 23;10:908864. doi: 10.3389/fpubh.2022.908864 (PMC9538358; doi:10.3389/fpubh.2022.908864)
Supplement: Supplementary file 1 [file Data_Sheet_1.docx]

**Supplementary Table 1 Summary of main data sources**

| Parameters | Value | Unit | Source |
| --- | --- | --- | --- |
| Insured population of urban employees | 32926 | ten thousand people | China Statistical Yearbook 2019 |
| Per capita GDP | 7.0892 | ten thousand yuan | China Statistical Yearbook 2019 |
| Insured population of urban and rural residents | 102482.7 | ten thousand people | China Statistical Yearbook 2019 |
| Total demand for nursing expenses per capita | 3 | ten thousand yuan | Survey data |
| Change rate of insured population of urban employees | 4.16% | Dimensionless | China Statistical Yearbook(2011-2019) |
| Change rate of insured population of urban and rural residents | 4.82% | Dimensionless | China Statistical Yearbook(2011-2019) |
| Change rate of per capita GDP | 10.54% | Dimensionless | China Statistical Yearbook(2011-2019) |
| Government per capita subsidy standard | 0.12% | Dimensionless | Assumed value |
| Change rate of per capita disposable income of urban residents | 9.64% | Dimensionless | China Statistical Yearbook(2011-2019) |
| enterprises payment rate | 0.12% | Dimensionless | Assumed value |
| Individual payment rate | 0. 08% | Dimensionless | Assumed value |
| Severe disability rate | 0.25% | Dimensionless | Historical literature[28] / Survey data |
| Reimbursement ratio | 70% | Dimensionless | Assumed value |


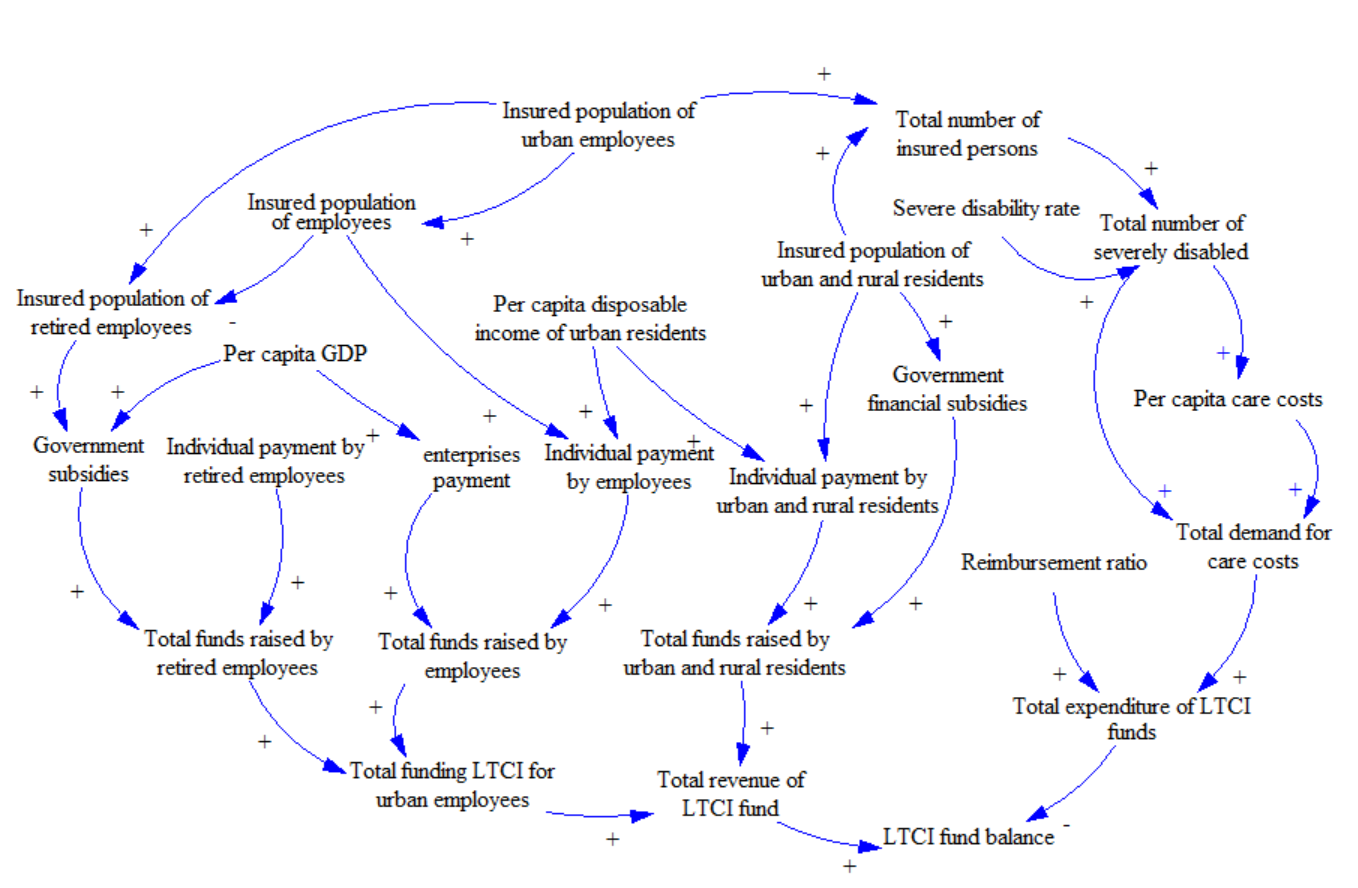


**Supplementary Figure 1 Causality diagram of LTCI financing system**


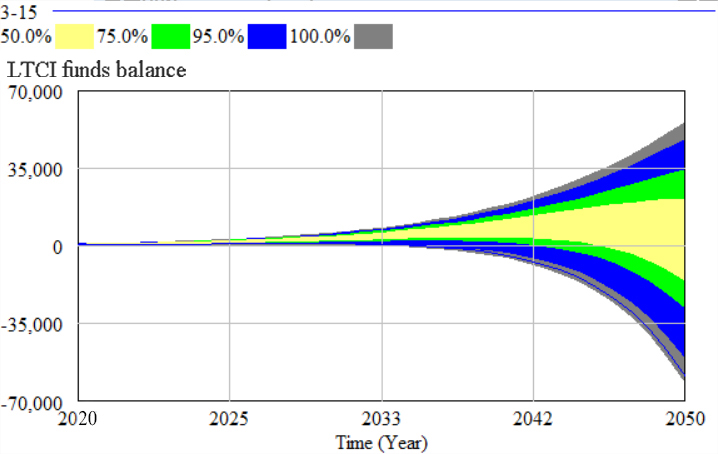


**Supplementary Figure 2 Sensitivity Analysis**

**Supplementary Table 2 Main function relation**

| **Variable** | **Functional formula** | **Unit** |
| --- | --- | --- |
| Insured population of employees | 135.446-0.733* Insured population of urban employees | ten thousand people |
| Insured population of retired employees | Insured population of urban employees - Insured population of employees | ten thousand people |
| enterprises payment | Per capita GDP*enterprises payment rate*Insured population of employees | hundred million |
| Individual payment by employees | Per capita disposable income of urban residents*Individual payment rate*Insured population of employees | hundred million |
| Individual payment by retired employees | Per capita disposable income of urban residents*Individual payment rate*Insured population of retired employees | hundred million |
| Government subsidies | Per capita GDP*Government per capita subsidy standard*Insured population of retired employees | hundred million |
| Total funds raised by employees | enterprises payment + Individual payment by employees | hundred million |
| Total funds raised by retired employees | Government subsidies + Individual payment by retired employees | hundred million |
| Total funds raised by urban employees | Total funds raised by employees + Total funds raised by retired employees | hundred million |
| Total funds raised by urban and rural residents | Individual payment by urban and rural residents + Government financial subsidies | hundred million |
| Total revenue of LTCI funds | Total funds raised by urban and rural residents + Total funds raised by urban employees | hundred million |
| Total demand for care costs | Per capita care costs*Reimbursement ratio | hundred million |
| Total expenditure of LTCI funds | Total number of severely disabled*Total demand for care costs | hundred million |
| Total number of insured persons | Insured population of urban and rural residents + Insured population of urban employees | ten thousand people |
| Total number of severely disabled | Total number of insured persons*Severe disability rate | ten thousand people |
| LTCI funds balance | Total revenue of LTCI funds-Total expenditure of LTCI funds | hundred million |

**Supplementary Table 3 Historical Test**

| Year | Medical insured population of urban employees | | |  | Per capita disposable income of urban residents | | |
| --- | --- | --- | --- | --- | --- | --- | --- |
|  | Actual data / ten thousand people | Simulation data / ten thousand people | Error rate /% |  | Actual data / ten thousand people | Simulation data / ten thousand people | Error rate /% |
| 2019 | 32926 | 34252.52 | 4.03 |  | 42359 | 42992.83 | 1.50 |
| 2018 | 31680.8 | 32884.52 | 3.80 |  | 39251 | 39212.72 | -0.10 |
| 2017 | 30322.7 | 31571.16 | 4.12 |  | 36396 | 35764.98 | -1.73 |
| 2016 | 29531.5 | 30310.25 | 2.64 |  | 33616 | 32620.37 | -2.96 |
| 2015 | 28893.1 | 29099.71 | 0.72 |  | 31195 | 29752.26 | -4.62 |
| 2014 | 28296 | 27937.51 | -1.27 |  | 28844 | 27136.32 | -5.92 |
| 2013 | 27443.4 | 26821.72 | -2.27 |  | 26467 | 24750.38 | -6.49 |
| 2012 | 26485.6 | 25750.50 | -2.78 |  | 24126.7 | 22574.22 | -6.43 |
| 2011 | 25227.1 | 24722.06 | -2.00 |  | 21426.9 | 20589.41 | -3.91 |

**Supplementary Table 4-1 When the individual payment rate is 0.08% and the severe disability rate is 0.25%**

|  | **0. 25%** | | | | |  | **0.25%** | | | | |
| --- | --- | --- | --- | --- | --- | --- | --- | --- | --- | --- | --- |
|  | **0.7** | **0.75** | **0.8** | **0.85** | **0.9** |  | **0.7** | **0.75** | **0.8** | **0.85** | **0.9** |
| **2019** | 899.9 | 849.1 | 798.3 | 747.6 | 696.8 | **2035** | 6984 | 6338 | 5693 | 5047 | 4401 |
| **2020** | 1026 | 966.4 | 906.9 | 847.3 | 787.8 | **2036** | 7903 | 7146 | 6390 | 5633 | 4876 |
| **2021** | 1169 | 1099 | 1030 | 959.9 | 890.1 | **2037** | 8938 | 8050 | 7163 | 6276 | 5388 |
| **2022** | 1332 | 1250 | 1168 | 1087 | 1005 | **2038** | 10,100 | 9060 | 8020 | 6979 | 5939 |
| **2023** | 1517 | 1421 | 1325 | 1229 | 1134 | **2039** | 11,400 | 10,190 | 8966 | 7746 | 6527 |
| **2024** | 1727 | 1615 | 1502 | 1390 | 1278 | **2040** | 12,870 | 11,440 | 10,010 | 8578 | 7148 |
| **2025** | 1966 | 1834 | 1702 | 1570 | 1439 | **2041** | 14,500 | 12,830 | 11,150 | 9476 | 7800 |
| **2026** | 2236 | 2082 | 1927 | 1773 | 1618 | **2042** | 16,340 | 14,370 | 12,410 | 10,440 | 8475 |
| **2027** | 2543 | 2362 | 2181 | 2000 | 1819 | **2043** | 18,380 | 16,070 | 13,770 | 11,470 | 9163 |
| **2028** | 2890 | 2678 | 2466 | 2253 | 2041 | **2044** | 20,660 | 17,950 | 15,250 | 12,550 | 9851 |
| **2029** | 3283 | 3035 | 2786 | 2537 | 2288 | **2045** | 23,190 | 20,020 | 16,850 | 13,690 | 10,520 |
| **2030** | 3729 | 3437 | 3145 | 2854 | 2562 | **2046** | 26,000 | 22,290 | 18,570 | 14,860 | 11,150 |
| **2031** | 4232 | 3890 | 3548 | 3206 | 2864 | **2047** | 29,110 | 24,760 | 20,410 | 16,050 | 11,700 |
| **2032** | 4801 | 4400 | 3999 | 3598 | 3198 | **2048** | 32,550^a^ | 27,450 | 22,340 | 17,240 | 12,140 |
| **2033** | 5443 | 4973 | 4503 | 4033 | 3564 | **2049** | 36,340^a^ | 30,360^a^ | 24,370 | 18,390 | 12,410 |
| **2034** | 6167 | 5617 | 5066 | 4515 | 3964 | **2050** | 40,500^a^ | 33,490^a^ | 26,470 | 19,460 | 12,440 |

Note. ^a^ refers to excessive funds balances.

**Supplementary Table 4-2 When the individual payment rate is 0.08% and the severe disability rate is 0.3%**

|  | **0.3%** | | | | | | | | | |  | | **0.3%** | | | | | | | |  |
| --- | --- | --- | --- | --- | --- | --- | --- | --- | --- | --- | --- | --- | --- | --- | --- | --- | --- | --- | --- | --- | --- |
|  | **0.7** | **0.75** | | **0.8** | | **0.85** | | **0.9** | | |  | | **0.7** | **0.75** | **0.8** | | **0.85** | | | **0.9** |  |
| **2019** | 757.7 | 696.8 | | 635.8 | | 574.9 | | 514 | | | **2035** | | 5176 | 4401 | 3626 | | 2852 | | | 2077 |  |
| **2020** | 859.2 | 787.8 | | 716.4 | | 645 | | 573.5 | | | **2036** | | 5784 | 4876 | 3967 | | 3059 | | | 2151 |  |
| **2021** | 973.8 | 890.1 | | 806.4 | | 722.7 | | 638.9 | | | **2037** | | 6453 | 5388 | 4324 | | 3259 | | | 2194 |  |
| **2022** | 1103 | 1005 | | 906.8 | | 808.6 | | 710.5 | | | **2038** | | 7187 | 5939 | 4691 | | 3442 | | | 2194 |  |
| **2023** | 1249 | 1134 | | 1019 | | 903.5 | | 788.5 | | | **2039** | | 7990 | 6527 | 5063 | | 3600 | | | 2136 |  |
| **2024** | 1412 | 1278 | | 1143 | | 1008 | | 873.1 | | | **2040** | | 8864 | 7148 | 5433 | | 3717 | | | 2001 |  |
| **2025** | 1597 | 1439 | | 1281 | | 1122 | | 964.4 | | | **2041** | | 9812 | 7800 | 5789 | | 3777 | | | 1766 |  |
| **2026** | 1804 | 1618 | | 1433 | | 1248 | | 1062 | | | **2042** | | 10,830 | 8475 | 6117 | | 3759 | | | 1401 |  |
| **2027** | 2036 | | 1819 | | 1601 | | 1384 | | 1167 | **2043** | | 11,930 | | 9163 | | 6399 | | 3634 | 869.4 | | |
| **2028** | 2296 | | 2041 | | 1787 | | 1532 | | 1277 | **2044** | | 13,090 | | 9851 | | 6610 | | 3369 | 127.8 | | |
| **2029** | 2587 | | 2288 | | 1990 | | 1691 | | 1393 | **2045** | | 14,320 | | 10,520 | | 6721 | | 2921 | -878.7^b^ | | |
| **2030** | 2912 | | 2562 | | 2212 | | 1862 | | 1512 | **2046** | | 15,600 | | 11,150 | | 6693 | | 2238 | -2217 ^b^ | | |
| **2031** | 3275 | | 2864 | | 2454 | | 2044 | | 1634 | **2047** | | 16,920 | | 11,700 | | 6477 | | 1254 | -3969 ^b^ | | |
| **2032** | 3679 | | 3198 | | 2717 | | 2236 | | 1755 | **2048** | | 18,260 | | 12,140 | | 6014 | | -109.8 ^b^ | -6234 ^b^ | | |
| **2033** | 4127 | | 3564 | | 3000 | | 2436 | | 1872 | **2049** | | 19,590 | | 12,410 | | 5227 | | -1952 ^b^ | -9132 ^b^ | | |
| **2034** | 4625 | | 3964 | | 3303 | | 2642 | | 1982 | **2050** | | 20,860 | | 12,440 | | 4024 | | -4394 ^b^ | -12,810 ^b^ | | |

Note. ^b^ refers to the funds balance deficit.

**Supplementary Table 4-3 When the individual payment rate is 0.08% and the severe disability rate is 0.35%**

|  | **0.35%** | | | | |  | **0.35%** | | | | |
| --- | --- | --- | --- | --- | --- | --- | --- | --- | --- | --- | --- |
|  | **0.7** | **0.75** | **0.8** | **0.85** | **0.9** |  | **0.7** | **0.75** | **0.8** | **0.85** | **0.9** |
| **2019** | 615.5 | 544.4 | 473.4 | 402.3 | 331.2 | **2035** | 3368 | 2464 | 1560 | 656.4 | -247.5 ^b^ |
| **2020** | 692.6 | 609.3 | 525.9 | 442.6 | 359.3 | **2036** | 3665 | 2605 | 1545 | 485.6 | -574.1 ^b^ |
| **2021** | 778.5 | 680.8 | 583.1 | 485.4 | 387.8 | **2037** | 3969 | 2726 | 1484 | 241.7 | -1001 ^b^ |
| **2022** | 874.1 | 759.6 | 645.1 | 530.6 | 416.1 | **2038** | 4275 | 2818 | 1362 | -94.57 ^b^ | -1551 ^b^ |
| **2023** | 980.2 | 846 | 711.8 | 577.5 | 443.3 | **2039** | 4575 | 2868 | 1161 | -546.9 ^b^ | -2254 ^b^ |
| **2024** | 1098 | 940.5 | 783.2 | 625.8 | 468.5 | **2040** | 4861 | 2859 | 857.5 | -1144 ^b^ | -3146 ^b^ |
| **2025** | 1228 | 1043 | 859 | 674.6 | 490.1 | **2041** | 5118 | 2772 | 424.9 | -1922 ^b^ | -4268 ^b^ |
| **2026** | 1371 | 1155 | 938.9 | 722.7 | 506.5 | **2042** | 5331 | 2580 | -171.5 ^b^ | -2923 ^b^ | -5674 ^b^ |
| **2027** | 1529 | 1275 | 1022 | 768.6 | 515.2 | **2043** | 5477 | 2252 | -973.7 ^b^ | -4199 ^b^ | -7424 ^b^ |
| **2028** | 1702 | 1405 | 1108 | 810.4 | 513.3 | **2044** | 5530 | 1748 | -2033 ^b^ | -5814 ^b^ | -9596 ^b^ |
| **2029** | 1890 | 1542 | 1194 | 845.6 | 497.3 | **2045** | 5455 | 1021 | -3412 ^b^ | -7845 ^b^ | -12,280 ^b^ |
| **2030** | 2096 | 1687 | 1279 | 870.7 | 462.4 | **2046** | 5208 | 10.43 | -5187 ^b^ | -10,380 ^b^ | -15,580 ^b^ |
| **2031** | 2318 | 1839 | 1360 | 881.7 | 403.1 | **2047** | 4736 | -1357 ^b^ | -7451 ^b^ | -13,540 ^b^ | -19,640 ^b^ |
| **2032** | 2556 | 1995 | 1434 | 873.3 | 312.2 | **2048** | 3973 | -3172 ^b^ | -10,320 ^b^ | -17,460 ^b^ | -24,600 ^b^ |
| **2033** | 2812 | 2154 | 1496 | 838.8 | 181 | **2049** | 2834 | -5542 ^b^ | -13,920 ^b^ | -22,290 ^b^ | -30,670 ^b^ |
| **2034** | 3083 | 2312 | 1541 | 769.9 | -1.135 ^b^ | **2050** | 1218 | -8602 ^b^ | -18,420 ^b^ | -28,240 ^b^ | -38,060 ^b^ |

Note. ^b^ refers to the funds balance deficit.

**Supplementary Table 5-1 When the individual payment rate is 0.09% and the severe disability rate is 0.25%**

|  | **0.25%** | | | | |  | **0.25%** | | | | |
| --- | --- | --- | --- | --- | --- | --- | --- | --- | --- | --- | --- |
|  | **0.7** | **0.75** | **0.8** | **0.85** | **0.9** |  | **0.7** | **0.75** | **0.8** | **0.85** | **0.9** |
| **2019** | 957.3 | 906.5 | 855.7 | 804.9 | 754.1 | **2035** | 7503 | 6857 | 6211 | 5566 | 4920 |
| **2020** | 1092 | 1032 | 972.7 | 913.2 | 853.6 | **2036** | 8499 | 7742 | 6985 | 6228 | 5471 |
| **2021** | 1245 | 1175 | 1105 | 1035 | 965.6 | **2037** | 9621 | 8734 | 7846 | 6959 | 6072 |
| **2022** | 1419 | 1337 | 1255 | 1173 | 1092 | **2038** | 10,880 | 9844 | 8804 | 7763 | 6723 |
| **2023** | 1617 | 1521 | 1425 | 1329 | 1233 | **2039** | 12,300 | 11,090 | 9866 | 8646 | 7426 |
| **2024** | 1841 | 1729 | 1617 | 1504 | 1392 | **2040** | 13,900 | 12,470 | 11,040 | 9611 | 8181 |
| **2025** | 2097 | 1965 | 1833 | 1701 | 1570 | **2041** | 15,690 | 14,010 | 12,340 | 10,660 | 8985 |
| **2026** | 2386 | 2232 | 2077 | 1923 | 1769 | **2042** | 17,700 | 15,730 | 13,770 | 11,800 | 9835 |
| **2027** | 2715 | 2534 | 2353 | 2172 | 1991 | **2043** | 19,940 | 17,640 | 15,330 | 13,030 | 10,720 |
| **2028** | 3088 | 2876 | 2664 | 2451 | 2239 | **2044** | 22,450 | 19,750 | 17,050 | 14,340 | 11,640 |
| **2029** | 3511 | 3262 | 3013 | 2764 | 2515 | **2045** | 25,240 | 22,080 | 18,910 | 15,740 | 12,580 |
| **2030** | 3989 | 3698 | 3406 | 3114 | 2823 | **2046** | 28,360 | 24,650 | 20,930 | 17,220 | 13,510 |
| **2031** | 4531 | 4189 | 3847 | 3505 | 3164 | **2047** | 31,820^a^ | 27,470 | 23,110 | 18,760 | 14,410 |
| **2032** | 5144 | 4743 | 4342 | 3942 | 3541 | **2048** | 35,660 ^a^ | 30,560 ^a^ | 25,450 | 20,350 | 15,250 |
| **2033** | 5837 | 5367 | 4897 | 4427 | 3958 | **2049** | 39,910 ^a^ | 33,920 ^a^ | 27,940 | 21,960 | 15,970 |
| **2034** | 6619 | 6069 | 5518 | 4967 | 4416 | **2050** | 44,600 ^a^ | 37,580 ^a^ | 30,570 ^a^ | 23,550 | 16,540 |

Note. ^a^ refers to excessive funds balances.

**Supplementary Table 5-2 When the individual payment rate is 0.09% and the severe disability rate is 0.3%**

|  | **0.3%** | | | | |  | **0.3%** | | | | |
| --- | --- | --- | --- | --- | --- | --- | --- | --- | --- | --- | --- |
|  | **0.7** | **0.75** | **0.8** | **0.85** | **0.9** |  | **0.7** | **0.75** | **0.8** | **0.85** | **0.9** |
| **2019** | 815.1 | 754.1 | 693.2 | 632.3 | 571.3 | **2035** | 5695 | 4920 | 4145 | 3370 | 2596 |
| **2020** | 925.1 | 853.6 | 782.2 | 710.8 | 639.4 | **2036** | 6379 | 5471 | 4563 | 3654 | 2746 |
| **2021** | 1049 | 965.6 | 881.9 | 798.2 | 714.5 | **2037** | 7136 | 6072 | 5007 | 3942 | 2877 |
| **2022** | 1190 | 1092 | 993.4 | 895.3 | 797.1 | **2038** | 7971 | 6723 | 5475 | 4226 | 2978 |
| **2023** | 1348 | 1233 | 1118 | 1003 | 887.9 | **2039** | 8890 | 7426 | 5963 | 4499 | 3036 |
| **2024** | 1527 | 1392 | 1257 | 1122 | 987.2 | **2040** | 9897 | 8181 | 6465 | 4750 | 3034 |
| **2025** | 1728 | 1570 | 1412 | 1253 | 1095 | **2041** | 11,000 | 8985 | 6974 | 4962 | 2951 |
| **2026** | 1954 | 1769 | 1583 | 1398 | 1213 | **2042** | 12,190 | 9835 | 7477 | 5119 | 2761 |
| **2027** | 2208 | 1991 | 1774 | 1557 | 1339 | **2043** | 13,490 | 10,720 | 7960 | 5195 | 2430 |
| **2028** | 2494 | 2239 | 1984 | 1730 | 1475 | **2044** | 14,880 | 11,640 | 8402 | 5161 | 1919 |
| **2029** | 2814 | 2515 | 2217 | 1918 | 1620 | **2045** | 16,380 | 12,580 | 8777 | 4977 | 1177 |
| **2030** | 3173 | 2823 | 2473 | 2123 | 1773 | **2046** | 17,960 | 13,510 | 9053 | 4598 | 142.6 |
| **2031** | 3574 | 3164 | 2753 | 2343 | 1933 | **2047** | 19,630 | 14,410 | 9186 | 3963 | -1261 |
| **2032** | 4022 | 3541 | 3060 | 2579 | 2098 | **2048** | 21,370 | 15,250 | 9122 | 2999 | -3125 |
| **2033** | 4521 | 3958 | 3394 | 2830 | 2266 | **2049** | 23,150 | 15,970 | 8795 | 1615 | -5565 |
| **2034** | 5077 | 4416 | 3755 | 3094 | 2434 | **2050** | 24,950 | 16,540 | 8119 | -299 | -8717 |

**Supplementary Table 5-3 When the individual payment rate is 0.09% and the severe disability rate is 0.35%**

|  | **0.35%** | | | | |  | **0.35%** | | | | |
| --- | --- | --- | --- | --- | --- | --- | --- | --- | --- | --- | --- |
|  | **0.7** | **0.75** | **0.8** | **0.85** | **0.9** |  | **0.7** | **0.75** | **0.8** | **0.85** | **0.9** |
| **2019** | 672.9 | 601.8 | 530.7 | 459.6 | 388.5 | **2035** | 3887 | 2983 | 2079 | 1175 | 271.2 |
| **2020** | 758.4 | 675.1 | 591.7 | 508.4 | 425.1 | **2036** | 4260 | 3200 | 2141 | 1081 | 21.21 |
| **2021** | 854 | 756.3 | 658.6 | 561 | 463.3 | **2037** | 4652 | 3409 | 2167 | 924.9 | -317.4 ^b^ |
| **2022** | 960.7 | 846.2 | 731.7 | 617.2 | 502.7 | **2038** | 5059 | 3602 | 2146 | 689.5 | -766.9 ^b^ |
| **2023** | 1080 | 945.4 | 811.2 | 677 | 542.8 | **2039** | 5475 | 3768 | 2060 | 352.9 | -1354 ^b^ |
| **2024** | 1212 | 1055 | 897.3 | 739.9 | 582.6 | **2040** | 5893 | 3892 | 1890 | -111.5 ^b^ | -2113 ^b^ |
| **2025** | 1359 | 1174 | 990 | 805.5 | 621.1 | **2041** | 6303 | 3957 | 1610 | -736.7 ^b^ | -3083 ^b^ |
| **2026** | 1522 | 1305 | 1089 | 872.9 | 656.7 | **2042** | 6691 | 3940 | 1189 | -1563 ^b^ | -4314 ^b^ |
| **2027** | 1701 | 1448 | 1194 | 941.1 | 687.6 | **2043** | 7038 | 3813 | 587.3 | -2638 ^b^ | -5864 ^b^ |
| **2028** | 1900 | 1603 | 1305 | 1008 | 711.2 | **2044** | 7321 | 3540 | -241.5 ^b^ | -4023 ^b^ | -7804 ^b^ |
| **2029** | 2117 | 1769 | 1421 | 1073 | 724.4 | **2045** | 7511 | 3077 | -1356 ^b^ | -5789 ^b^ | -10,220 ^b^ |
| **2030** | 2356 | 1948 | 1540 | 1131 | 723.1 | **2046** | 7568 | 2370 | -2827 ^b^ | -8025 ^b^ | -13,220 ^b^ |
| **2031** | 2617 | 2138 | 1659 | 1181 | 702.2 | **2047** | 7445 | 1351 | -4743 ^b^ | -10,840 ^b^ | -16,930 ^b^ |
| **2032** | 2900 | 2339 | 1778 | 1216 | 655.4 | **2048** | 7081 | -63.34 ^b^ | -7208 ^b^ | -14,350 ^b^ | -21,500 ^b^ |
| **2033** | 3206 | 2548 | 1890 | 1233 | 574.9 | **2049** | 6402 | -1975 ^b^ | -10,350 ^b^ | -18,730 ^b^ | -27,100 ^b^ |
| **2034** | 3535 | 2764 | 1993 | 1222 | 450.9 | **2050** | 5313 | -4508 ^b^ | -14,330 ^b^ | -24,150 ^b^ | -33,970 ^b^ |

Note. ^b^ refers to the funds balance deficit.

**Supplementary Table 6-1 When the individual payment rate is 0.1% and the severe disability rate is 0.25%**

|  | **0.25%** | | | | |  | **0.25%** | | | | |
| --- | --- | --- | --- | --- | --- | --- | --- | --- | --- | --- | --- |
|  | **0.7** | **0.75** | **0.8** | **0.85** | **0.9** |  | **0.7** | **0.75** | **0.8** | **0.85** | **0.9** |
| **2019** | 1015 | 963.8 | 913.1 | 862.3 | 811.5 | **2035** | 8021 | 7376 | 6730 | 6084 | 5439 |
| **2020** | 1158 | 1098 | 1039 | 979 | 919.5 | **2036** | 9094 | 8337 | 7580 | 6823 | 6066 |
| **2021** | 1320 | 1250 | 1181 | 1111 | 1041 | **2037** | 10,300 | 9417 | 8529 | 7642 | 6755 |
| **2022** | 1505 | 1424 | 1342 | 1260 | 1178 | **2038** | 11,670 | 10,630 | 9588 | 8547 | 7507 |
| **2023** | 1716 | 1620 | 1524 | 1428 | 1332 | **2039** | 13,200 | 11,980 | 10,770 | 9546 | 8326 |
| **2024** | 1955 | 1843 | 1731 | 1618 | 1506 | **2040** | 14,930 | 13,500 | 12,070 | 10,640 | 9214 |
| **2025** | 2227 | 2096 | 1964 | 1832 | 1701 | **2041** | 16,880 | 15,200 | 13,520 | 11,850 | 10,170 |
| **2026** | 2537 | 2382 | 2228 | 2073 | 1919 | **2042** | 19,060 | 17,090 | 15,130 | 13,160 | 11,200 |
| **2027** | 2888 | 2707 | 2526 | 2344 | 2163 | **2043** | 21,500 | 19,200 | 16,890 | 14,590 | 12,290 |
| **2028** | 3286 | 3074 | 2861 | 2649 | 2437 | **2044** | 24,240 | 21,540 | 18,840 | 16,140 | 13,430 |
| **2029** | 3738 | 3489 | 3240 | 2991 | 2743 | **2045** | 27,300 | 24,130 | 20,970 | 17,800 | 14,630 |
| **2030** | 4250 | 3958 | 3667 | 3375 | 3083 | **2046** | 30,720 ^a^ | 27,010 | 23,290 | 19,580 | 15,870 |
| **2031** | 4830 | 4488 | 4146 | 3804 | 3463 | **2047** | 34,530 ^a^ | 30,180 ^a^ | 25,820 | 21,470 | 17,120 |
| **2032** | 5487 | 5086 | 4686 | 4285 | 3884 | **2048** | 38,770 ^a^ | 33,660 ^a^ | 28,560 | 23,460 | 18,350 |
| **2033** | 6231 | 5761 | 5291 | 4821 | 4351 | **2049** | 43,470 ^a^ | 37,490 ^a^ | 31,510 ^a^ | 25,520 | 19,540 |
| **2034** | 7071 | 6521 | 5970 | 5419 | 4868 | **2050** | 48,690 ^a^ | 41,670 ^a^ | 34,660 ^a^ | 27,650 | 20,630 |

Note. ^a^ refers to excessive funds balances.

**Supplementary Table 6-2 When the individual payment rate is 0.1% and the severe disability rate is 0.3%**

|  | **0.3%** | | | | |  | **0.3%** | | | | |
| --- | --- | --- | --- | --- | --- | --- | --- | --- | --- | --- | --- |
|  | **0.7** | **0.75** | **0.8** | **0.85** | **0.9** |  | **0.7** | **0.75** | **0.8** | **0.85** | **0.9** |
| **2019** | 872.4 | 811.5 | 750.6 | 689.6 | 628.7 | **2035** | 6213 | 5439 | 4664 | 3889 | 3114 |
| **2020** | 990.9 | 919.5 | 848 | 776.6 | 705.2 | **2036** | 6975 | 6066 | 5158 | 4250 | 3341 |
| **2021** | 1125 | 1041 | 957.4 | 873.7 | 790 | **2037** | 7820 | 6755 | 5690 | 4625 | 3560 |
| **2022** | 1276 | 1178 | 1080 | 982 | 883.8 | **2038** | 8755 | 7507 | 6259 | 5010 | 3762 |
| **2023** | 1448 | 1332 | 1217 | 1102 | 987.4 | **2039** | 9790 | 8326 | 6863 | 5399 | 3936 |
| **2024** | 1641 | 1506 | 1371 | 1236 | 1101 | **2040** | 10,930 | 9214 | 7498 | 5782 | 4067 |
| **2025** | 1859 | 1701 | 1542 | 1384 | 1226 | **2041** | 12,180 | 10,170 | 8159 | 6148 | 4136 |
| **2026** | 2104 | 1919 | 1734 | 1548 | 1363 | **2042** | 13,550 | 11,200 | 8837 | 6479 | 4121 |
| **2027** | 2381 | 2163 | 1946 | 1729 | 1512 | **2043** | 15,050 | 12,290 | 9521 | 6756 | 3991 |
| **2028** | 2692 | 2437 | 2182 | 1928 | 1673 | **2044** | 16,680 | 13,430 | 10,190 | 6952 | 3711 |
| **2029** | 3041 | 2743 | 2444 | 2146 | 1847 | **2045** | 18,430 | 14,630 | 10,830 | 7033 | 3233 |
| **2030** | 3433 | 3083 | 2733 | 2383 | 2033 | **2046** | 20,320 | 15,870 | 11,410 | 6957 | 2502 |
| **2031** | 3873 | 3463 | 3052 | 2642 | 2232 | **2047** | 22,340 | 17,120 | 11,890 | 6671 | 1448 |
| **2032** | 4365 | 3884 | 3403 | 2922 | 2441 | **2048** | 24,480 | 18,350 | 12,230 | 6107 | -16.88 |
| **2033** | 4915 | 4351 | 3788 | 3224 | 2660 | **2049** | 26,720 | 19,540 | 12,360 | 5183 | -1997 |
| **2034** | 5529 | 4868 | 4207 | 3546 | 2886 | **2050** | 29,050 | 20,630 | 12,210 | 3796 | -4622 |

**Supplementary Table 6-3 When the individual payment rate is 0.09% and the severe disability rate is 0.35%**

|  | **0.35%** | | | | |  | **0.35%** | | | | |
| --- | --- | --- | --- | --- | --- | --- | --- | --- | --- | --- | --- |
|  | **0.7** | **0.75** | **0.8** | **0.85** | **0.9** |  | **0.7** | **0.75** | **0.8** | **0.85** | **0.9** |
| **2019** | 730.3 | 659.2 | 588.1 | 517 | 445.9 | **2035** | 4406 | 3502 | 2598 | 1694 | 790 |
| **2020** | 824.2 | 740.9 | 657.6 | 574.2 | 490.9 | **2036** | 4855 | 3796 | 2736 | 1676 | 616.5 |
| **2021** | 929.5 | 831.9 | 734.2 | 636.5 | 538.8 | **2037** | 5335 | 4093 | 2850 | 1608 | 365.7 |
| **2022** | 1047 | 932.9 | 818.4 | 703.9 | 589.4 | **2038** | 5843 | 4386 | 2930 | 1473 | 17.1 |
| **2023** | 1179 | 1045 | 910.7 | 776.4 | 642.2 | **2039** | 6375 | 4667 | 2960 | 1253 | -454.7 ^b^ |
| **2024** | 1326 | 1169 | 1011 | 854.1 | 696.7 | **2040** | 6926 | 4924 | 2923 | 921.1 | -1081 ^b^ |
| **2025** | 1490 | 1305 | 1121 | 936.5 | 752 | **2041** | 7488 | 5142 | 2795 | 448.5 | -1898 ^b^ |
| **2026** | 1672 | 1456 | 1239 | 1023 | 807 | **2042** | 8051 | 5300 | 2549 | -202.4 ^b^ | -2954 ^b^ |
| **2027** | 1874 | 1620 | 1367 | 1114 | 860.1 | **2043** | 8599 | 5374 | 2148 | -1077 ^b^ | -4303 ^b^ |
| **2028** | 2097 | 1800 | 1503 | 1206 | 909.1 | **2044** | 9113 | 5331 | 1550 | -2231 ^b^ | -6013 ^b^ |
| **2029** | 2345 | 1996 | 1648 | 1300 | 951.5 | **2045** | 9567 | 5133 | 700.1 | -3733 ^b^ | -8167 ^b^ |
| **2030** | 2617 | 2208 | 1800 | 1392 | 983.7 | **2046** | 9928 | 4730 | -467.7 ^b^ | -5665 ^b^ | -10,860 ^b^ |
| **2031** | 2916 | 2437 | 1958 | 1480 | 1001 | **2047** | 10,150 | 4059 | -2034 ^b^ | -8128 ^b^ | -14,220 ^b^ |
| **2032** | 3243 | 2682 | 2121 | 1560 | 998.6 | **2048** | 10,190 | 3045 | -4099 ^b^ | -11,240 ^b^ | -18,390 ^b^ |
| **2033** | 3600 | 2942 | 2284 | 1626 | 968.8 | **2049** | 9969 | 1593 | -6783 ^b^ | -15,160 ^b^ | -23,540 ^b^ |
| **2034** | 3987 | 3216 | 2445 | 1674 | 902.9 | **2050** | 9407 | -413.3 ^b^ | -10,230 ^b^ | -20,050 ^b^ | -29,870 ^b^ |

Note. ^b^ refers to the funds balance deficit.
